# Case of Melanosis of the Stomach

**Published:** 1826-07

**Authors:** 


MELANOSE OF THE STOMACH.—ANDRALS, JUN.

A woman, 50 years of age, died at La Charité under M. Lerminier, in February, 1825. From the time of her reception, she had had general serous infiltrations of the cellular tissue, as well as ascites. The cause of this was sought for in vain. The action of the heart was regular—no appearance of disease in the liver, nor in any other organ. The history given by the patient was unsatisfactory. The dropsy had commenced gradually, first as anasarca, and then ascites. She never had pain in the abdomen. She was six weeks in the hospital, during which the dropsy did not diminish, and the debility increased. Diarrhœa obtained from time to time, and anorexia was complete. No vomiting, no epigastric pain—tongue natural. The patient sunk in the most gradual manner, without presenting any other phenomena.

*Dissection.* No disease of the heart, lungs, liver, or thoracic duct, which was carefully traced. On opening the stomach, a quantity of liquid as black as ink escaped, amounting to three glassfuls (small tumblers.) On washing out the stomach, its internal surface was found studded with circular or oval spots, of a jet black colour. Some of these were as large as a franc piece, the others much smaller, and indeed of all sizes down to that of a millet seed. Around the larger patches, the mucous membrane appeared inflamed and almost livid, these appearances diminishing in proportion as the distance from the spots was increased. Otherwise the internal surface of the stomach was pale. The seat of this melanosis was entirely confined to the mucous membrane. There was nothing remarkable in the other portions of the digestive canal.

M. Andral thinks it proper to publish this case, 1^mo. Because very few examples of such deposition of pigmentum nigrum in the form of circular patches are on record, as affecting the stomach. 2^ndo. Because this case is not without interest, in a medico-legal point of view, as presenting lesions which might be taken for those produced by certain poisons, as sulphuric acid. 3^tio. Because this case proves, that a matter analogous to that of the black vomit of people affected with stomach cancer may, in some cases, be exhaled from this organ, without the existence of any cancerous affection, or even of gastritis. He thinks it probable, and in this we agree with him, that the disease in question was of a very recent formation, and that the black exhalation was in small quantity before death, and consequently did not excite vomiting.

The only other circumstance worthy of remark, in this case, was the absence of all organic disease to account for the dropsy. Our author could only attribute it therefore to a defect in the equilibrium between the exhâlents and absorbents—to too great activity in the one, or too little in the other. In fact, we are often unable to attribute the cause of dropsy to any other than this unequilibrium between the two systems, whatever theories may be spun out on the occasion. In such a case as that now presented there is every reason to attribute the dropsical effusion to debility of the absorbents rather than to any excess of action in the exhalent vessels. The rage for generalising, at the present moment, makes dropsy always dependent on increased exhalation. Dr. Parry, of Bath, gave the greatest force and development to this doctrine, in his Elements of Pathology; but, with all deference to his commanding genius and unbounded experience, we cannot help considering him as having carried the doctrine too far. Errare est humanum!—*Archives, Mars.*

---

